# Endoscopic diagnosis of sessile serrated polyp: A systematic review

**DOI:** 10.1111/den.13263

**Published:** 2018-09-30

**Authors:** Hiroshi Kashida

**Affiliations:** ^1^ Department of Gastroenterology and Hepatology Kindai University Osaka Japan

**Keywords:** differential diagnosis, endoscopy, sessile serrated adenoma, sessile serrated polyp, sessile serrated polyp/adenoma

## Abstract

The aim of the present review was to clarify how we should detect and diagnose sessile serrated polyps (SSP) endoscopically. A systematic search was conducted of MEDLINE from January 2004 through March 2018. Nine findings: (i) proximal location; (ii) size >10 mm; (iii) irregular shape; (iv) indistinctive border; (v) cloud‐like surface; (vi) mucus cap; (vii) rim of debris in white‐light endoscopy; (viii) dilated vessels; and (ix) dilated crypts (pits) in image‐enhanced endoscopy were considered to be candidate discriminators of SSP from hyperplastic polyps. Prospective studies in a general setting are warranted to validate the above‐mentioned endoscopic features of SSP during real‐time colonoscopy and to determine whether these features are useful for the differential diagnosis of SSP.

## Introduction

More than 20 years have passed since Longacre *et al*.,^1^ Torlakovic *et al*.^2^ and Snover *et al*.^3^ reported on serrated lesions in the colorectum which are now divided into hyperplastic polyp, traditional serrated adenoma, and sessile serrated adenoma/polyp (SSA/P) with or without dysplasia by the WHO classification system.^4^ Sessile serrated adenoma/polyp is considered to be an important precursor of colorectal cancer which remains one of the leading causes of cancer deaths worldwide.^5^ However, Payne *et al*.^6^ described that the term “serrated” never appeared in pathology reports at 10 of 32 centers. There is significant interobserver variation among pathologists in identifying and classifying serrated lesions.^7^, ^8^, ^9^, ^10^, ^11^ Even the terminology for serrated lesions may be subject to change. In this article, the terms conventional adenomatous polyp (AP), traditional serrated adenoma (TSA), sessile serrated polyp (SSP) and hyperplastic polyp (HP) will be used.

There is a recent tendency that the detection rate of SSP is increasing.^12^, ^13^ Reasons for this phenomenon are attributed not only to the use of new endoscopic techniques but also to the increased awareness of endoscopists and pathologists concerning the significance and appearance of SSP. Detection rates of SSP are also known to be endoscopist‐dependent.^14^, ^15^, ^16^, ^17^ The true prevalence of SSP is not evident due to lack of knowledge, difficulty in detection and unestablished diagnostic criteria. Among others, endoscopic discrimination between SSP and HP remains a problem. The role of advanced imaging techniques (IEE) such as narrow‐band imaging (NBI) in recognition of these lesions is also not well established. Use of an appropriate classification system including IEE would facilitate the recognition of SSP. The aim of the present review was to clarify specific characteristics and defining features that can help distinguish SSP from HP.

## Search Design and Keywords

**Figure 1:**
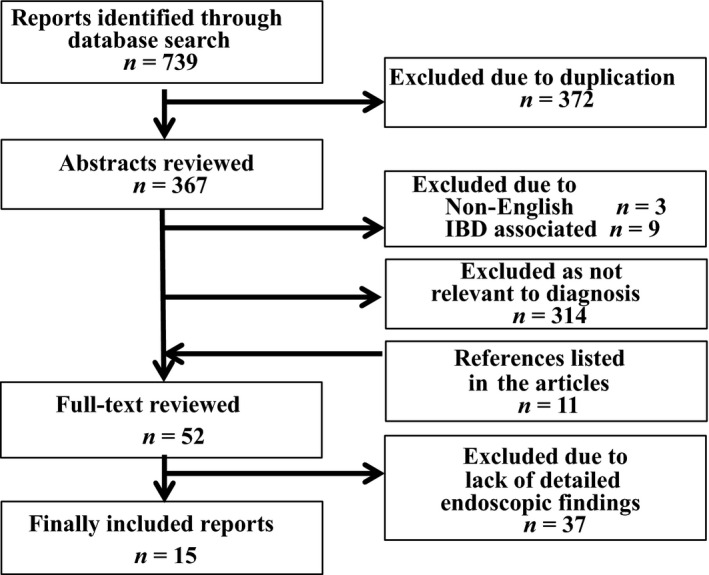
Flow diagram of study selection. IBD, irritable bowel disease.

For the purpose of the present review, a systematic search was carried out in Medical Literature Analysis and Retrieval System On‐Line (MEDLINE) from January 2004 through March 2018. Keywords included endoscopy, characteristics, detection, and diagnosis. Each keyword was paired with the SSA/P‐related terms (serrated lesion, sessile serrated adenoma, sessile serrated polyp, sessile serrated adenoma/polyp, SSA, SSP, and SSA/P). Articles on the endoscopic characteristics of SSP were included (Fig. 1). Non‐English articles were excluded. If the abstract seemed to meet the inclusion criteria, the full text was reviewed. Articles on serrated lesions in inflammatory bowel disease were excluded. References listed in the articles were also evaluated manually in order to identify any additional study. If a citation was believed to potentially meet the inclusion criteria, the full article was then reviewed. Finally, 15 original papers were selected and reviewed in the present study (Table 1).^18^, ^19^, ^20^, ^21^, ^22^, ^23^, ^24^, ^25^, ^26^, ^27^, ^28^, ^29^, ^30^, ^31^, ^32^

**Table 1:** Study design of the articles included in the present study

Reference/First author	Study design	No. of SSP	Modality	Evidence level
Hasegawa^18^	Retrospective cohort, single center	22	WLE, chromo, zoom	Low
Kashida^19^	Retrospective cohort, single center	35	HR‐WLE, chromo, zoom	Moderate
Yamada^20^	Retrospective cohort, single center	118	NBI, zoom	
	Validation study, 3 experts	19		Moderate
Yamada^21^	Retrospective cohort, single center	57	WLE, chromo	Moderate
Ishigooka^22^	Retrospective cohort, single center	50	HR‐WLE, chromo, zoom	Moderate
Tadepalli^23^	Retrospective cohort, single center	158	WLE, NBI	Moderate
Bouwens^24^	Retrospective cohort, single center	170	HR‐WLE, i‐scan, chromo	Moderate
Lee^25^	Retrospective cohort, single center	634	HR‐WLE	Low
Kawasaki^26^	Retrospective cohort, single center	80	HR‐WLE, NBI, chromo, zoom	Low
Hazewinkel^27^	Retrospective cohort, single center	50	HR‐WLE, NBI	Moderate
IJspeert^28^	Validation study, 10 experts	First 15	HR‐WLE, NBI	High
		Second 11		
Nakao^29^	Retrospective cohort, single center	46	HR‐WLE, NBI, zoom	Low
Uraoka^30^	Prospective, multicenter	38	HR‐WLE, NBI, chromo, zoom	High
Yamashina^31^	Retrospective cohort, single center	39	NBI, zoom	
	Validation study, 12 experts	51		High
Kimura^32^	Retrospective and	Training 69	HR‐WLE, chromo, zoom	
	prospective, single center	Validation 55		High

HR‐WLE, high‐resolution white‐light endoscopy; NBI, narrow‐band imaging; SSP, sessile serrated polyp.

## Findings in white‐light endoscopy

Hasegawa *et al*.^18^ conducted an investigation on 107 sessile serrated lesions (31 32 TSA, 22 SSP and 54 HP) in order to clarify endoscopic features of sessile serrated lesions and to determine whether SSP can be distinguished from other serrated lesions. Mean lesion size of SSP was larger than that of HP (14.2 *vs* 6.2 mm; *P* < 0.01). SSP was located more often in the proximal colon than in the distal colon (81.8% *vs* 18.2%), whereas HP was located less often in the proximal colon than in the distal colon (44.4% *vs* 55.6%). Limitation of this study is the small number of subjects and the retrospective nature.

Kashida *et al*.^19^ found 673 (6.0%) serrated lesions out of 11 253 colorectal localized lesions that were resected in their institute. Among them, those >5 mm (232 TSA, 35 SSP, and 95 HP) were evaluated. Average size of lesion was substantially bigger in SSP than in HP (14.0 ± 7.1 *vs* 7.9 ± 3.6 mm, *P* < 0.001). SSP were mostly located in the proximal colon, whereas the majority of HP were located in the distal colon.

Yamada *et al*.^20^ conducted a multivariate analysis among 242 lesions (118 SSP and 124 HP) on the clinical features of SSP compared with HP. They found that proximal location, size ≥10 mm, and sessile morphology were independent features of SSP, with odds ratio (OR) (95% CI) of 18.3 (7.2–46.2), 26.6 (10.6–66.4) and 4.3 (1.8–10.2), respectively.

Yamada *et al*.^21^ retrospectively examined 202 serrated lesions of the colorectum (41 TSA, 57 SSP and 104 HP). SSP were more predominantly located in the proximal colon and significantly larger than HP (*P* < 0.01). Polyp surface was granular or nodular in 40% of the SSP, more frequently than in HP (*P* < 0.01). Margins were more irregular or vague in SSP than in HP (*P* = 0.02). “Mucus‐coated appearance” was observed in nine of 57 SSP, but only in one of 104 HP (*P* < 0.01).

Ishigooka *et al*.^22^ investigated 112 lesions including 39 TSA, 50 SSP and 23 HP. SSP were larger in diameter than HP (13.62 ± 8.62 *vs* 7.74 ± 3.24 mm), more commonly located in the proximal colon (84.0% *vs* 30.4%), flat‐elevated (90.0% *vs* 30.4%), orthocolor or pale (80% *vs* 34.8%), and with a larger amount of mucus (72.0% *vs* 21.7%).

Tadepalli *et al*.^23^ retrospectively analyzed 158 proximal SSP in 124 patients, 47.1% of which were larger than 10 mm in diameter. By Paris Classification,^33^ all lesions were type 0; 1.9% were protruded 0‐Is, 96% were slightly elevated (0‐IIa), and 4% were truly flat (0‐IIb). They evaluated the prevalence of seven morphological findings in 158 SSP and in 40 AP. Prevalent characteristics of SSP were mucus cap (63.9%), rim of debris/bubbles (51.9%), alteration of fold contour (37.3%), and obscured blood vessels (32.3%). In the review of 69 videos with 69 SSP and 31AP, the most frequent keys to the detection of SSP were mucus cap (24.6%), alteration of fold contour (24.6%), and rim of debris/bubbles (21.7%). Some of the most frequent findings such as alteration of fold contour were witnessed in both SSP and AP; therefore, these findings cannot be used as discriminators between SSP and AP. Moreover, it would be distracting to discuss this point further when the main purpose of the present study is discrimination between SSP and HP.

Bouwens *et al*.^24^ retrospectively examined 170 SSP based on Tadepalli's criteria.^23^ The lesions showed obscured blood vessels in 54.7%, alteration of fold contour in 5.3%, rim of debris/bubbles in 40.5%, mucus cap in 32.6% and pale color in 19.4%. The major aim of the study was to clarify endoscopic characteristics of SSP with dysplasia but there was no significant difference of endoscopic appearance between those with and without dysplasia except that dome‐shaped elevation was more often witnessed in the former.

## Serrated Lesions Mimicking Diverticula

Lee *et al*.^25^ reported a group of SSP with a depressed surface. They accounted for seven out of 634 SSP. Kawasaki *et al*.^26^ found similar lesions in seven out of 80 SSP and in two out of 35 HP. The present author has also encountered many such cases of SSP. Whether or not these lesions with depressed surface are a subgroup of SSP is yet to be established. However, we should be aware of this type as there is a potential risk of misdiagnosing them as colonic diverticula.

These lesions look similar to so‐called “inverted hyperplastic polyps” in previous reports,^34^, ^35^, ^36^, ^37^, ^38^ most of which had been published before the concept of SSP became popular and well‐recognized. Presence of colonic crypts in submucosa has been also termed as herniation of crypts, pseudoinvasion, or epithelial‐misplacement. Hu *et al*.^39^ conducted a prospective pathological investigation. Among 2560 colorectal polyps, frequencies of herniation of crypts were 1.79% (10/559) in SSP, 0.2% (3/1487) in HP and 0% (0/514) in polypoid normal tissue. Although the specificity of herniation of crypts for diagnosing SSP (*vs* HP and polypoid normal tissue) was 99.85% (1998/2001), its sensitivity was only 1.79% (10/559). Their multivariate analyses identified an independent association between herniation of crypts and diagnosis of SSP (OR = 11.47, *P* = 0.009 for *vs* HP). Note that diverticulum‐mimicking SSP may not necessarily be associated with the presence of crypts in the submucosal layer.

The present author has also encountered many such cases of SSP. There is no convincing evidence to conclude that “diverticulum‐mimicking appearance” is a feature of SSP versus HP because the number of study subjects was small and the overall incidence of this finding was low. However, the incidence might be underestimated as a result of insufficient awareness of the phenomenon by most endoscopists.

## Narrow‐Band Imaging Without Magnification

In the study conducted by Rex *et al*.,^40^ 800 patients were randomized to the NBI arm and to the white light (WL) arm. The authors concluded that NBI may increase the detection of proximal serrated lesions, although the result in this study was not statistically significant.

Boparai *et al*.^41^ examined seven patients with serrated polyposis syndrome (SPS) using high‐resolution (HR) WL and NBI. In total, subjects had 15 AP, 32 SSP and 19 HP. Endoscopic differentiation between AP and HP was possible with the surface pattern and vessel pattern in NBI, but that between SSP and HP was unsatisfactory.

In the study by Tadepalli *et al*.,^23^ NBI was carried out in 62 SSP. In 43 (69.4%) SSP, NBI enhanced the visibility of SSP compared with WL. The enhancing effect of NBI was most frequently noticed in the SSP with mucus cap (38/45, 84.4%), those with rim of debris (31/37, 83.8%) and those with obscured blood vessels (17/22, 77.3%).

In the study by Hazewinkel *et al*.,^27^ HR‐WLE and NBI pictures of 150 polyps (50 AP, 50 SSP and 50 HP) in 45 patients with SPS were systematically assessed using seven potential endoscopic features of SSP as follows: indistinctive border, cloud‐like surface, dark spots inside the crypts, irregular shape, absence of tiny microvessels crossing the surface, pit pattern II SSA/P (mixture of open crypts and small elongated star‐shaped pits), and vascular pattern intensity (VPI).

Multivariate analysis showed that indistinctive borders (OR, 3.11; 95% CI, 1.57–6.15) and cloud‐like surface (OR, 2.65; 95% CI, 1.21–5.78) were independent predictive findings of SSP in HR‐WLE. In NBI cloud‐like surface (OR, 4.91; 95% CI, 2.42–9.97), irregular shape (OR, 3.17; 95% CI, 1.59–6.29), indistinctive borders (OR, 2.38; 95% CI, 1.14–4.96), and dark spots inside the crypts (OR, 2.05; 95% CI, 1.02–4.11) were independent predictive factors for SSP.

A lesion was presumed to be SSP if all of six SSP features were present, whereas it was considered to be HP if all of these features were absent. Using these conditions, sensitivity and specificity were 75% and 79% in HR‐WLE and 89% and 96% in NBI, respectively. In their study, only serrated polyps that showed either all or no independent features were compared.

IJspeert *et al*.^28^ developed the Workgroup serrAted polypS and Polyposis (WASP) classification. The polyps were first evaluated using the NBI International Colorectal Endoscopic (NICE) criteria in order to divide them into HP‐like lesions (Type 1 polyps) and those resembling AP (Type 2 polyps). Secondly Hazewinkel's criteria for SSP^27^ were used to differentiate between SSP and HP for Type 1 polyps, and between SSP and AP for Type 2 polyps. Presence of at least two out of four SSP features was considered sufficient for the diagnosis of SSP.

Ten consultant gastroenterologists were asked to participate in the training. Location and size of polyps were not presented. Overall accuracy was 0.63 before training, which increased significantly to 0.79 after training (16% improvement; 95% CI: 9–22%; *P* < 0.001). In the polyps diagnosed with high confidence, overall accuracy significantly increased from 0.73 to 0.87 (14% improvement; 95% CI 4–24%; *P* < 0.01).

## Narrow‐Band Imaging with Magnification

Nakao *et al*.^29^ studied 46 SSP and 25 HP with magnifying endoscopy with NBI (M‐NBI). Frequency of the findings with M‐NBI was as follows: capillary dilatation: 11% in SSP and 4% in HP; mucus cap: 94% in SSP and 60% in HP; and pit dilatation: 80% in SSP and 28% in HP. In the distinction of SSP from HP, sensitivity and specificity were 10% and 96% for capillary dilatation, 94% and 40% for mucus cap, and 80% and 72% for pit dilatation, respectively.

Uraoka *et al*.^30^ investigated 89 lesions including 38 SSP (median size: 17 mm), and 41 HP (median size: 11 mm). They evaluated: (i) location; (ii) size; (iii) macroscopic type; (iv) adherent mucus; (v) brownish area in non‐magnified NBI (N‐NBI); (vi) “varicose microvascular vessel (VMV)” in magnified NBI (M‐NBI); (vii) depression in chromoendoscopy (indigocarmine); (viii) “expanded Type II (E‐II) pit pattern”; and (ix) “IIIH pit pattern”, using high‐magnification chromoendoscopy. Multivariate analysis showed that three factors: “VMV”, size ≥10 mm, and proximal location, were independent diagnostic factors for SSP with OR of 8.2 (*P* = 0.001), 7.2 (*P* = 0.0017), and 6.1 (*P* = 0.0041), respectively. Sensitivity and specificity of “VMV” were 87.8% and 57.9%, respectively. If a lesion was considered SSP when two or more criteria were positive, sensitivity was 89.5% and overall accuracy was 82.3%.

Yamashina *et al*.^31^ conducted a pilot study with M‐NBI and detected brownish, oval, “expanded crypt openings (ECO)” and “thick and branched vessels (TBV)” as diagnostic features of SSP. They then prospectively validated the criteria in 125 polyps with NICE Type 1 (51 SSP and 74 others) with an average size of 4–7 mm. Sensitivity and specificity of ECO for SSP were 84.3% and 81.1%, whereas those of TBV were 45.1% and 68.9%, respectively. If a lesion presumed to be SSP when ECO or TBV or both were present, the sensitivity and specificity were 98% and 59.5%, respectively.

Yamada *et al*.^20^ conducted a study using M‐NBI and selected five features: dilated and branching vessels (DBV), irregular dark spots (iDS), regular network pattern, disorganized network pattern, and dense pattern as potential M‐NBI features of SSP. Multivariate analysis among 242 lesions estimated that DBV only was the criterion for discriminating SSP from HP. They then systematically validated the diagnostic accuracies by using the pictures of 40 lesions (19 SSP and 21 HP). Sensitivity and specificity of DBV were 79% and 67%, respectively, whereas those of iDS were 42% and 81%, respectively. Assuming that a lesion is SSP when both DBV and iDS were present, the sensitivity, specificity and OR (95% CI) by univariate analysis were 26%, 95% and 7.14 (0.75–68.0), respectively. Assuming that a lesion is SSP when either of DBV or iDS was present, the sensitivity, specificity and OR (95% CI) were 95%, 52% and 19.8 (2.22–176.6), respectively.

## Chromoendoscopy with Magnification

Kimura *et al*.^32^ identified a pit pattern that was specific to SSP and named this “Type II‐open (Type II‐O)”. Pits with this type were similar to conventional Type II pits, but were larger and more rounded in shape, reflecting the dilated crypts. The authors hypothesized that an overproduction of mucin was the cause of this phenotype. Next, they prospectively analyzed an independent validation set (*n* = 116; 61 AP and 55 serrated lesions). The results showed that “Type II‐O pattern” was highly predictive of SSP (sensitivity: 65.5% and specificity: 97.3%).

In the study by Ishigooka *et al*.,^22^ magnified colonoscopy showed “Type II‐O pit pattern” as characteristic of SSP (sensitivity: 83.7% and specificity: 85.7%). The pinecone‐like pit pattern was characteristic of TSA (sensitivity: 96.7% and specificity: 89.9%). Note that Uraoka *et al*.^30^ expressed this pit pattern as “expanded Type II (E‐II) pit pattern”.

## Discussion

It would be better to discuss the detection of SSP and its diagnosis separately; however, this was impossible because almost none of the reviewed articles did so. Actually, it is very difficult to completely separate these two processes in a real‐world clinical setting; for example, interruption of mucosal capillary network can be a key to detect a flat lesion, but it is not a good discriminator among AP, HP and SSP. Mucus cap is a key to detect a serrated lesion and, at the same time, it is a key to diagnose an SSP.

Other endoscopic technologies such as Full Spectrum Endoscopy (FUSE) ^®^(EndoChoice, Inc, Alpharetta, GA, US) or ENDOCUFF™ (Arc Medical Design Ltd, Leeds, UK) have been attempted to improve the detection rate of SSP, but these are out of the range of the present article. I excluded the literature that discussed this point as it is related to improvement of polyp detection in general and is not specific for SSP.

**Figure 2:**
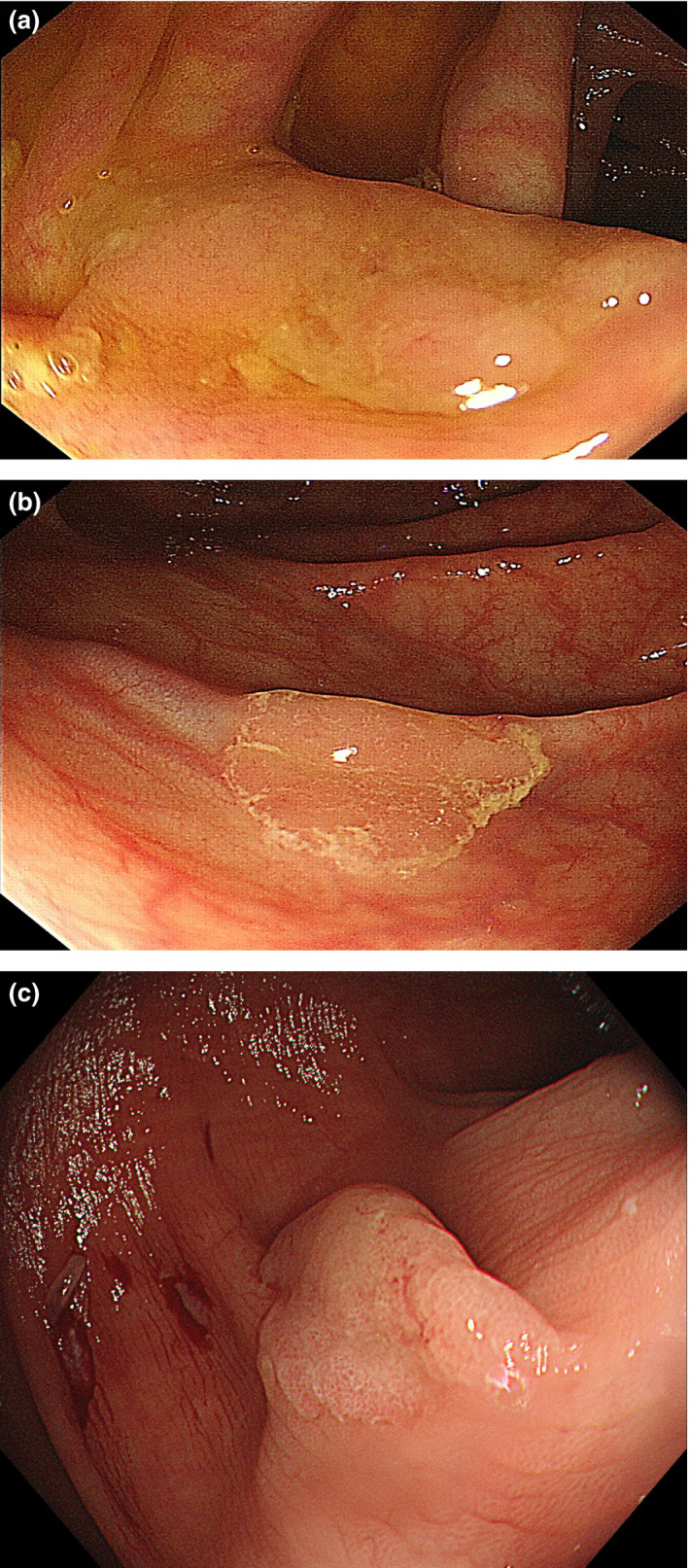
White light images of sessile serrated polyp. (a) An irregular‐shaped lesion with cloud‐like surface and mucus cap. (b) A flat lesion with a rim of debris. (c) A sessile lesion with an irregular shape, pale in color.

**Figure 3:**
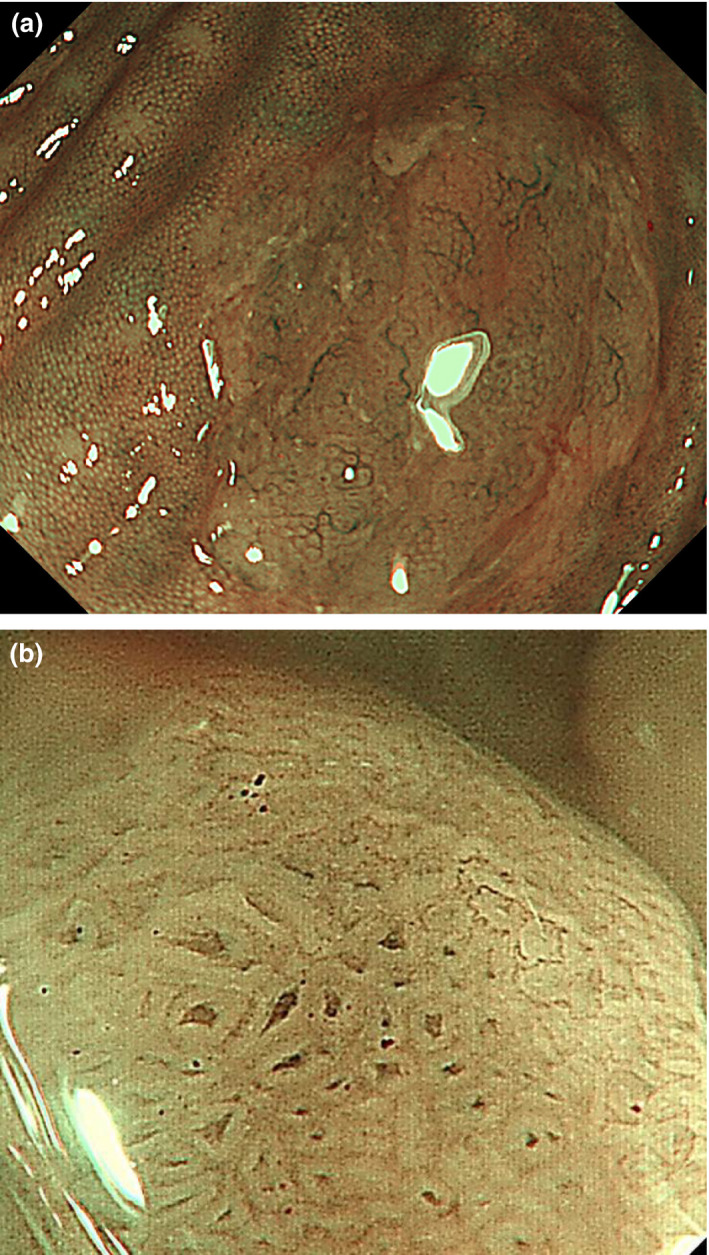
Narrow‐band imaging (NBI) of sessile serrated polyp (SSP). (a) Non‐magnified NBI. Color of the flat‐elevated lesion is the same as the surrounding mucosa. Several dilated vessels can be identified. (b) Magnified NBI. Several dilated crypts are evident.

**Figure 4:**
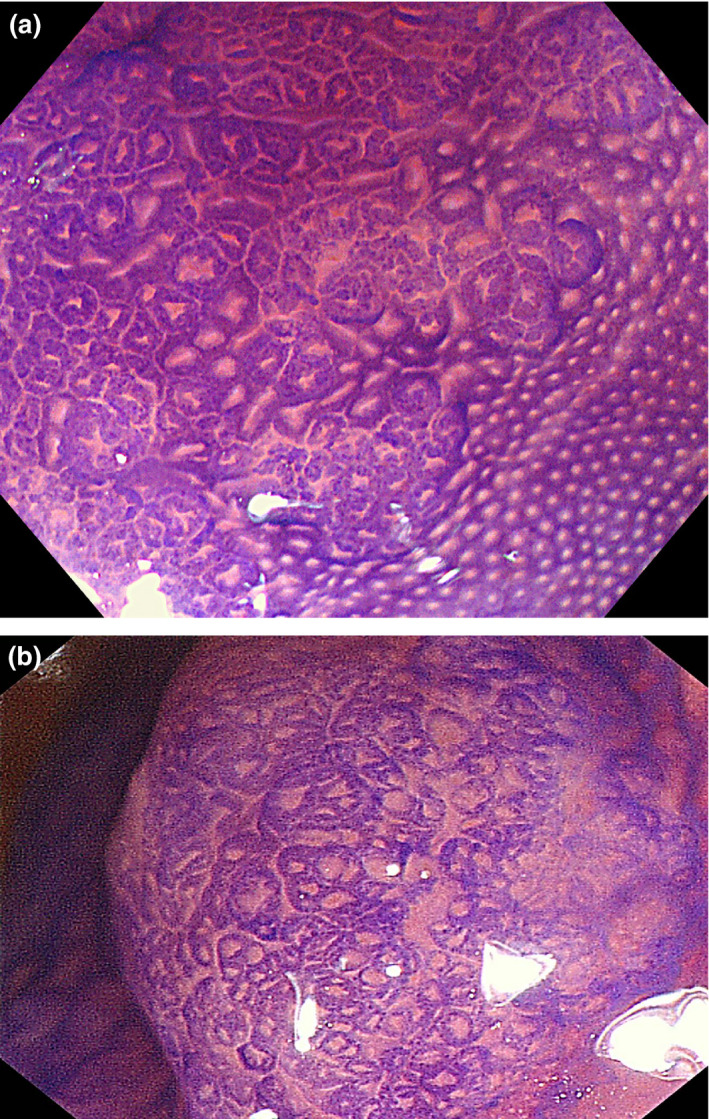
Chromoendoscopic views of sessile serrated polyp. (a,b) Magnified view after crystal violet staining. The pits are larger than those of the surrounding normal mucosa. Some of them are dilated and round, but there are also star‐like pits.

**Figure 5:**
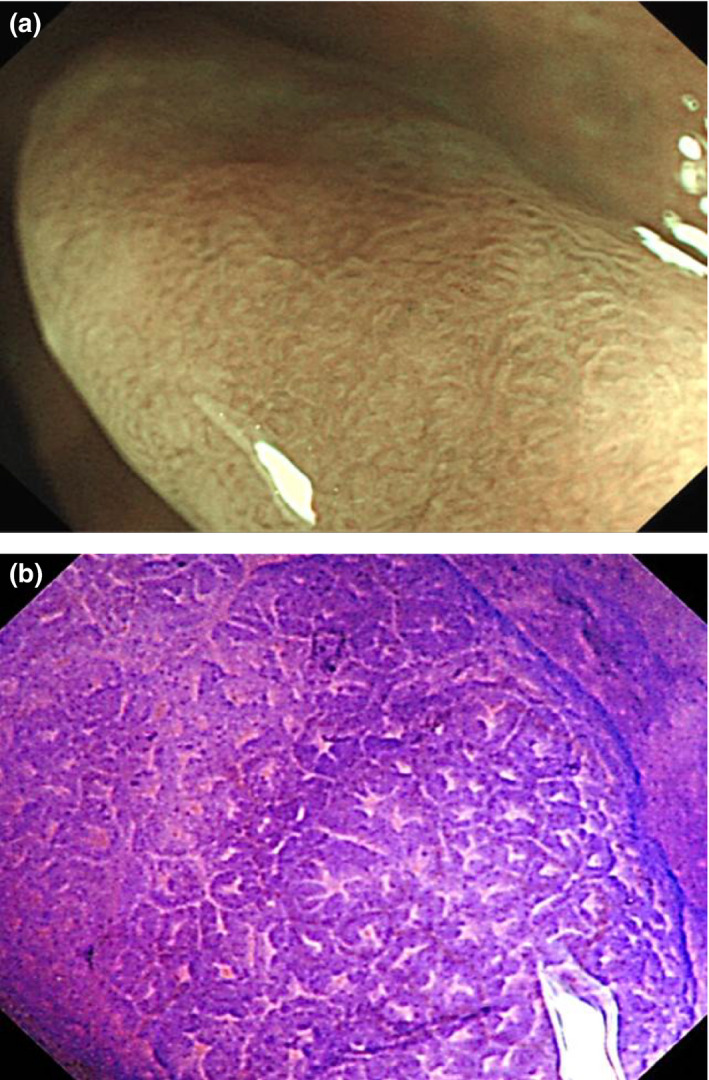
Endoscopic views of hyperplastic polyp. (a) Magnified narrow‐band imaging. Vessels or pits are hardly recognized. (b) Magnified view after crystal violet staining. Pits are star‐shaped.

Endoscopic characteristics of HP and SSP are summarized in Table 2.Endoscopic findings: (i) proximal location; (ii) size > 10 mm; (iii) irregular shape; (iv) indistinctive border; (v) cloud‐like surface; (vi) mucus cap; (vii) rim of debris; (viii) dilated vessels; and (ix) dilated crypts (pits) are candidate discriminators of SSP from HP (Figs 2, 3, 4, 5).

**Table 2:** Endoscopic characteristics of HP and SSP

	HP	SSP
White light		
Location	Distal	Proximal^a^
Size	Usually <5 mm	Can be >10 mm^b^
Shape	Flat‐elevated oval, circular	Flat‐elevated or sessile irregular^c^
Border	Indistinctive‐distinctive	Indistinctive^d^
Surface	Smooth	Cloud‐like^e^ Diverticulum‐like Mucus cap^f^ Rim of debris^g^
Color	Pale	Normal or pale
NBI		
Vessel	None or lacy	None or dilated^h^
Surface pattern	None Dark or white spots	Dilated crypts^i^
Chromoendoscopy		
Pit	Star‐shaped (Type II)	Dilated round pits^i^ or fern‐like

^a–i^Candidate discriminators of sessile serrated polyps (SSP) from hyperplastic polyps (HP).^e^A cloud‐like surface is also expressed as a granular, nodular, or bumpy surface and looks like the surface of a cumulus cloud.^h^A dilated vessel is also expressed as a thick and branched vessel (TBV), dilated and branching vessel (DBV) or varicose microvascular vessel (VMV).^i^Dilated crypts seen with narrow‐band imaging (NBI) are also expressed as dark spots inside the crypts, pit dilatation, expanded crypt openings (ECO), or irregular dark spots (iDS). Dilated round pits seen with magnified chromoendoscopy are expressed as Type II‐open (Type II‐O) or expanded Type II (Type E‐II). It is considered that dilated crypts seen with NBI and dilated round pits seen with magnified chromoendoscopy reflect the same feature of SSP and can be used as synonyms.

SSP can be small or diminutive. However, when a flat and pale‐color lesion is more than 10 mm in diameter, it is more likely to be an SSP than a HP. A cloud‐like surface is also expressed as a granular, nodular, or bumpy surface and looks like the surface of a cumulus cloud. A dilated vessel is also expressed as a TBV, DBV or VMV. Vessels are not prominent in SSP, but when a non‐pericryptal, dilated tortuous vessel is present, the lesion is more likely to be an SSP than a HP. HP can rarely present with some vessels on the surface, but they are usually only lacy.

Dilated crypts seen with NBI are also expressed as dark spots inside the crypts, pit dilatation, ECO, or iDS. Dilated round pits seen with magnified chromoendoscopy are expressed as Type II‐O or Type E‐II. It is considered that dilated crypts seen with NBI and dilated round pits seen with magnified chromoendoscopy reflect the same feature of SSP and can be used almost as synonyms. Even in SSP it is not that all the pits are dilated, but only some of the pits are round and open. They are usually interspersed with star‐like (Type II) pits or fern‐like pits. This finding with endoscopy corresponds to the pathological finding of SSP; most of the crypts are serrated with some of the crypts filled with mucus and dilated.

A meta‐analysis of 13 studies carried out by Parikh *et al*.^42^ concluded that image‐enhanced endoscopy cannot be recommended as a diagnostic tool for SSP. However, this meta‐analysis encompassed different modalities of image‐enhanced endoscopy including auto‐fluorescence imaging (AFI). They described that NBI studies showed promise. More studies are needed to validate the NBI criteria for diagnosing SSP. Likewise, more investigations are required to prove the usefulness of new modalities such as Blue Laser Imaging, i‐scan.

Recognition of dysplasia within SSP^43^ is another important concern, but it is out of the range of the present study. It requires another review article.

Prospective studies during real‐time colonoscopy in an ordinary clinical setting among a large number of subjects are warranted to confirm the above‐mentioned endoscopic features of SSP and to validate whether these features are useful for the detection and differential diagnosis of SSP.

## Conflicts of Interest

Authors declare no conflicts of interest for this article.
